# Comparative genomics reveals diverse capsular polysaccharide synthesis gene clusters in emerging *Raoultella planticola*

**DOI:** 10.1590/0074-02760180192

**Published:** 2018-08-27

**Authors:** Yao-Ting Huang, Wei-Yao Chuang, Bing-Ching Ho, Zong-Yen Wu, Rita C Kuo, Mengwei Ko, Po-Yu Liu

**Affiliations:** 1 National Chung Cheng University, Department of Computer Science and Information Engineering, Chia-Yi, Taiwan; 2 National Taiwan University Hospital, Department of Clinical Laboratory Sciences and Medical Biotechnology, Taipei, Taiwan; 3 US Department of Energy Joint Genome Institute, Walnut Creek, CA, USA; 4 National Chung Hsing University, Department of Veterinary Medicine, Taichung, Taiwan; 5 University of California, Division of Oral Biology and Oral Medicine, School of Dentistry and Medicine, The Jane and Jerry Weintraub Center for Reconstructive Biotechnology, Los Angeles, CA, USA; 6 Shu-Zen Junior College of Medicine and Management, Department of Nursing, Kaohsiung City, Taiwan; 7 National Chung Hsing University, College of Life Sciences, Rong Hsing Research Center for Translational Medicine, Taichung, Taiwan; 8 National Chung Hsing University, PhD Program in Translational Medicine, Taichung, Taiwan; 9 Taichung Veterans General Hospital, Department of Internal Medicine, Division of Infectious Diseases, Taichung, Taiwan

**Keywords:** Raoultella planticola, carbapenem resistance, capsular polysaccharide

## Abstract

*Raoultella planticola* is an emerging zoonotic pathogen that is associated with rare but life-threatening cases of bacteremia, biliary tract infections, and urinary tract infections. Moreover, increasing antimicrobial resistance in the organism poses a potential threat to public health. In spite of its importance as a human pathogen, the genome of *R. planticola* remains largely unexplored and little is known about its virulence factors. Although lipopolysaccharides has been detected in *R. planticola* and implicated in the virulence in earlier studies, the genetic background is unknown. Here, we report the complete genome and comparative analysis of the multidrug-resistant clinical isolate *R. planticola* GODA. The complete genome sequence of *R. planticola* GODA was sequenced using single-molecule real-time DNA sequencing. Comparative genomic analysis reveals distinct capsular polysaccharide synthesis gene clusters in *R. planticola* GODA. In addition, we found *bla*_TEM_-57 and multiple transporters related to multidrug resistance. The availability of genomic data in open databases of this emerging zoonotic pathogen, in tandem with our comparative study, provides better understanding of *R. planticola* and the basis for future work.

*Raoultella species* are facultative anaerobic gram-negative bacilli found in plants, wood, soil, water, and wildlife.^1^ The genus contains four species: *Raoultella planticola*,^2^ *Raoultella electrica*,^3^ *Raoultella ornithinolytica*,^4^ and *Raoultella terrigena*.^5^ *R. planticola* is the most common human pathogen in the genus, causing biliary tract infections and urinary tract infections.^6^

The vast majority of patients infected with *R. planticola* are immunocompromised individuals such as such as organ transplant recipients and those with malignancy or diabetes mellitus.^7^ Recently, there are increasing reports of severe cases presented with bacteremia and sepsis.^6^ Moreover, increasingly resistant strains of *R. planticola* have emerged and are responsible for the majority of health-care-associated infections.^1^ ^,^ ^8^ Study also revealed the organism is capable to survive in a range of hospital environments by developing resistance to disinfectants.^9^

Genetic analysis is essential in successfully addressing emerging infectious diseases.^10^ ^,^ ^11^ Although a few *R. planticola* genome sequences are available, the genomic background of its pathogenesis and resistance is largely unknown. Here, we sequenced and reconstructed the complete circular genome of the *R. planticola* strain GODA and performed genome-wide comparisons in order to decipher the putative virulence and resistance determinants.

*R. planticola* GODA strain was isolated from the blood sample of a septic patient. Antimicrobial susceptibility testing using automated Vitek 2 system (bioMérieux, Marcy-l’Étoile, France) revealed that the organism was resistant to multiple antibiotics. *R. planticola* GODA was resistant to cefazolin (MIC ≥ 64 μg/mL), ceftriaxone (MIC ≥ 64 μg/mL), ceftazidime (MIC ≥ 64 μg/mL), cefepime (MIC ≥ 64 μg/mL), ampicillin/sulbactam (MIC ≥ 32 μg/mL), piperacillin/tazobactam (MIC ≥ 128 μg/mL), trimethoprim/ sulfamethoxazole (MIC ≥ 320 μg/mL), and imipenem (MIC = 4 μg/mL) and susceptible to amikacin (MIC < 2 μg/mL) and ciprofloxacin (MIC = 1 μg/mL).

*R. planticola* GODA was grown in Luria-Bertani broth overnight at 37ºC. The overnight culture (1 to 5 × 10^8^ CFU/mL) was pelleted and resuspended in PBS. Genomic DNA was extracted with DNeasy blood and tissue kit (Qiagen, Hilden, Germany), following the manufacturer’s instructions. DNA was sheared to 10kb using the g-TUBE™ (Covaris). The sheared DNA was treated with DNA damage repair mix followed by end repair and ligation of SMRT adapters using the PacBio SMRTbell Template Prep Kit (Pacific Biosciences, Menlo Park, CA, United States). Whole genome sequencing was performed using the PacBio sequencing platform (Pacific Biosciences). Sequence runs of three single-molecule real-time (SMRT) cells were performed on the PacBio RS II sequencer with a 120-minute movie time/SMRT cell. SMRT Analysis portal version 2.1 was used for read filtering and adapter trimming, with default parameters, and post-filtered data of 1.2Gb (around 214X coverage) with an average read length of 6 kb were used for subsequent assembly.

The post-filtered reads were de novo assembled by Canu (v1.4) and converted into circular form via Circlator. These long reads were assembled and circularized into a complete circular genome (~5.6Mbp). Meanwhile, three additional plasmids were also reconstructed. The guanine-cytosine (GC) content of the GODA genome was 55.4%, which was similar with other related strains. Protein-coding genes in the genome and plasmids were annotated using NCBI Prokaryotic Genomes Automatic Annotation Pipeline (PGAAP). Functional classification of annotated genes was carried out by RPSBLAST v. 2.2.15 in conjunction with the COGs (Clusters of Orthologous Groups of proteins) database. A total of 5,461 genes were identified, including 25 rRNA genes, and 83 tRNA genes (Table I).

We further constructed a pan-genome dataset using whole genome sequence of GODA and 7 publicly available whole genome sequences of *R. planticola* strains (Table I). We considered each gene to be strain-specific if it was present only in one strain and absent in all other strains. Furthermore, the genes shared by all strains were considered to be pan-genomic core genes. Fig. 1 shows orthologous genes shared among strains and depicts the position and color-coded function of the *R. planticola* GODA-specific genes. The numbers of orthologous and strain-specific unique genes are shown in the Venn diagrams (Fig. 2A). As presented in the figure, the pan genome of *R. planticola* revealed 4,382 core genes shared across all strains, whereas 147 genes were specific to *R. planticola* GODA. Functional analysis of GODA-specific genes revealed that, in addition to hypothetical proteins, a relative abundance of these gene are involved in replication and repair, followed by cell wall/membrane/envelop biogenesis (Fig. 2B). The Average Nucleotide Identity (ANI) was calculated based on a modified algorithm^12^ and revealed that *R. planticola* GODA is closely related to ATCC 33531, FDAARGOS_64, and CHB in terms of nucleotide sequences (ANI > 98%) (Fig. 3).

Virulence genes in the GODA genome were identified using the virulence factor database (VFDB). The identified virulence genes, which were also GODA-specific genes, were considered to be putative GODA-specific virulence factors.

The polysaccharide capsule is considered a major virulence factor of *R. planticola* (formerly named *Klebsiella planticola*).^13^ Previous study in *Klebsiella* spp. suggests the *wzx* is a common component in the capsular polysaccharide biosynthesis pathway.^14^ Our comparative genomics also revealed the presence of *wzx* flippase in the GODA genome, but this was lost in the environmental strains. Further investigation of its upstream and downstream genes revealed the entire capsular polysaccharide synthesis (cps) gene cluster (Fig. 4). Our findings provide the first genetic background of the cps gene clusters in *R. planticola*.

We further compared the cps clusters of environmental/clinical isolated strains and two distant-related *Klebsiella* strains (Fig. 4). Three highly conserved genes: *galF*, *gnd* and *ugd* were well-preserved across all strains analyzed, whereas the gene composition in between was often variable. A similar context has been noted in *Klebsiella* strains.^15^ The inter-species variability (*R. planticola* vs *Klebsiella* strains) was relatively higher than the intra-species variability. The cps structure of two clinical isolates, GODA and FDAARGOS_64, were highly similar, implying both strains may express identical virulence factors. While *wzx* was commonly found in *Klebsiella* spp., it was lost in all environmental isolated strains of *R. planticola* in this study.^14^

Genetic context analysis of the capsular polysaccharide synthesis gene cluster of GODA showed that *wzx* was located between a gene encoding UTP--glucose-1-phosphate uridylyltransferase and a 6-phosphogluconate dehydrogenase. A similar observation has been made in several capsular polysaccharide synthesis gene clusters of *Klebsiella* spp.^14^ Capsular polysaccharide is a major virulence factor of *Klebsiella* spp. and genetic structures of the capsular polysaccharide synthesis gene cluster in *Klebsiella* spp. have been well studied.^16^ Generally, *galF* at the 5’ end of the capsular polysaccharide regions and *gnd* and *ugd* at the 3’ end are highly conserved among different *Klebsiella*. The same context was identified in GODA. We also predicted genes encoding proteins necessary for capsular polysaccharide translocation and processing at the cell surface (*wza*, *wzb*, *wzc*, and *wzi*) and genes encoding glycosyltransferase.

**TABLE I:** Features of *Raoultella planticola* strains in the study

Strain	Site of isolation	Country of origin	Genome assembly status	Genome size (bp)	GC content (%)	CDSs (pseudo genes)	rRNA operons	tRNAs
GODA	Human	Taiwan	Complete	5,592,163	55.4	5,461(703)	25	83
ATCC 33531	Radish root	Unknown	Contig	5,668,028	55.8	5,363(193)	5	67
CHB	River	USA	Contig	5,780,876	55.4	5,501(210)	24	77
FDAARGOS_64	Human	USA	Contig	5,823,731	55.6	5,541(312)	25	86
1175_2058	Human	USA	Contig	5,750,464	55.7	5,486(96)	19	71
626_SENT	Human	USA	Scaffold	5,735,751	55.5	5,544(233)	3	28
INSali127	Vegetable	Portugal	Scaffold	6,011,051	55.5	5,843(211)	5	72
INSali133	Vegetable	Portugal	Scaffold	6,011,836	55.5	5,840(220)	5	74

CDSs: coding sequences; GC: guanine-cytosine.

**Fig. 1:**
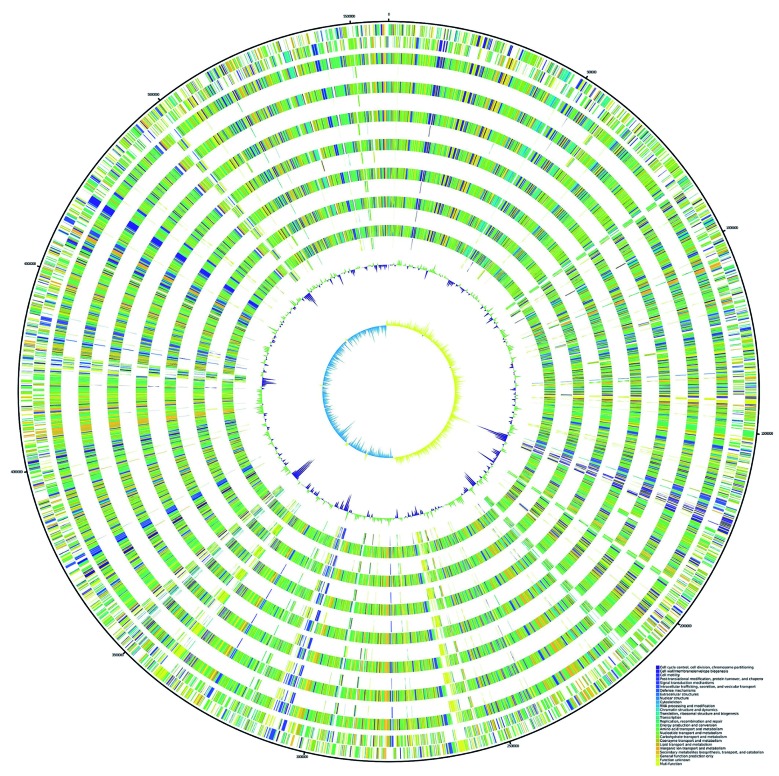
circular genomes representation map and genome comparison of *Raoultella planticola* (GODA, 1175_2058, 626_SENT, ATCC 33531, CHB, FDAARGOS_64, INSali127, INSali133). Predicted coding sequences (CDSs) are assigned various colors with respect to cellular functions. Circles show from the outermost to the innermost: (1) DNA coordinates; (2, 3). Function-based color-coded mapping of the CDSs predicted on the forward and reverse strands of the *R. planticola* GODA genome, respectively; (4) Orthologous CDSs shared between *R. planticola* GODA and *R. planticola* 1175_2058; (5) *R. planticola* GODA-specific CDSs, compared with *R. planticola* 1175_2058; (6) Orthologous CDSs shared between *R. planticola* GODA and *R. planticola* 626_SENT; (7) *R. planticola* GODA-specific CDSs, compared with *R. planticola* 626_SENT; (8) Orthologous CDSs shared between *R. planticola* GODA and *R. planticola* ATCC 33531; (9) *R. planticola* GODA-specific CDSs, compared with *R. planticola* ATCC 33531; (10) Orthologous CDSs shared between *R. planticola* GODA and *R. planticola* CHB; (11) *R. planticola* GODA-specific CDSs, compared with *R. planticola* CHB; (12) Orthologous CDSs shared between *R. planticola* GODA and *R. planticola* FDAARGOS_64; (13) *R. planticola* GODA-specific CDSs, compared with *R. planticola* FDAARGOS_64; (14) Orthologous CDSs shared between *R. planticola* GODA and *R. planticola* INSali127; (15) *R. planticola* GODA-specific CDSs, compared with *R. planticola* INSali127; (16) Orthologous CDSs shared between *R. planticola* GODA and *R. planticola* INSali133; (17) *R. planticola* GODA-specific CDSs, compared with *R. planticola* INSali133; (18) GC plot with regions above and below average in green and violet; (19) GC skew showing regions above and below average in yellow and light blue. This figure was plotted in Scalable Vector Graphics format via an in-house script, which calculates the radius and ribbon width according to the BLAST alignments and adds colors by COG classification of all orthogonal genes.

**Fig. 2:**
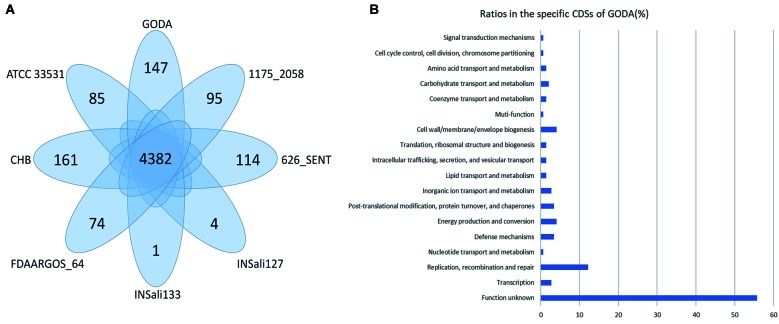
comparison of the gene contents of the *Raoultella planticola*. (A) Venn diagram showing the numbers of conserved and strain-specific coding sequences (CDSs). 4,382 core genes shared across all strains, whereas 147 genes were specific to *R. planticola* GODA. (B) COG category-based functional analysis of GODA-specific CDSs. This figure was constructed using Microsoft PowerPoint.

**Fig. 3:**
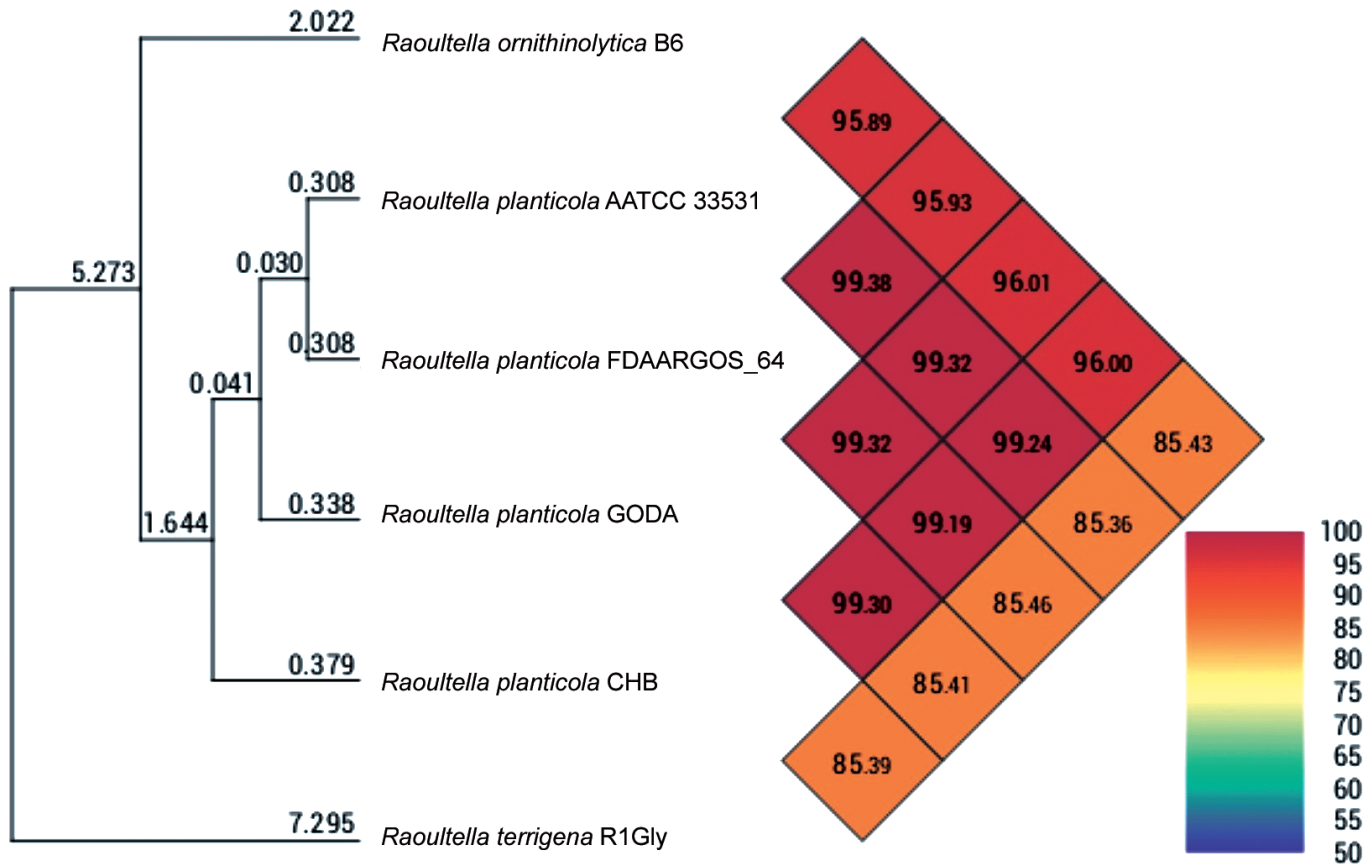
heat-map of average nucleotide identity values between each genome of *Raoultella planticola* strains and related species. *R. planticola* GODA is closely related to ATCC 33531, FDAARGOS_64, and CHB. This figure was depicted by OrthoANI (https://www.ezbiocloud.net/tools/orthoani).

The resistome in GODA was annotated using the Resistance Gene Identifier from the Comprehensive Antibiotic Resistance Database (CARD)^17^ and IMG database^18^. GODA showed the presence of *bla*_TEM_-57 (Table II), an extended-spectrum β-lactamase conferring resistance against β-lactam antibiotics such as penicillins and cephalosporins.^19^ GODA was also equipped with a number of efflux systems. GODA contains homologs of multidrug and toxic compound extrusion (MATE) family (*mdtK*), resistance-nodulation-cell division (RND) family (*mdtABC*, *oqxAB*, *acrAB*), ATP (adenosine triphosphate)-binding cassette (ABC) superfamily (*yojI*, *msbA*), and major facilitator superfamily (MFS) efflux pump (*emrAB*, *mdtL*, *rosAB*). These multidrug-resistance efflux pumps, along with or in combination with extended-spectrum β-lactamase could result in resistance to multiple classes of antibiotics.^20^ ^,^ ^21^

**Fig. 4:**
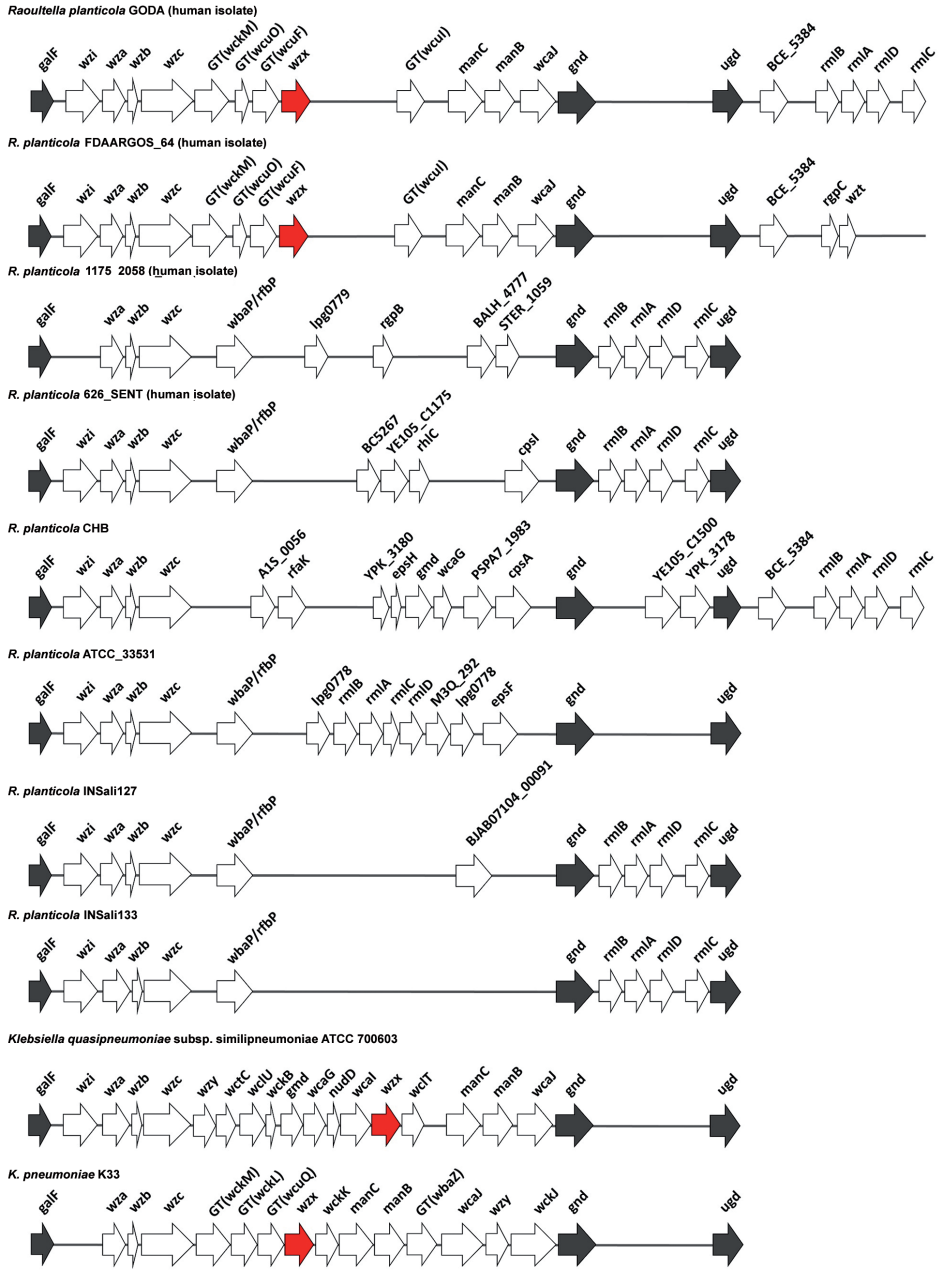
genomic comparison of the cps gene cluster in *Raoultella planticola* reveals genetic diversity. Gene clusters are shown in gray. Strain specific *wzx* genes are marked in red color. GT: glycosyltransferase. This figure was constructed using Microsoft PowerPoint.

**TABLE II:** Phenotypic resistance profile and putative resistance determinant in strain GODA

Antimicrobial agents (Subclasses)	MIC (µg/mL)	Interpretation	Putative resistance determinant
β-lactams			*bla*_TEM-57_
Ampicillin/sulbactam	≥ 32	R	
Piperacillin/tazobactam	≥ 128	R	
Cefazolin	≥ 64	R	
Cefoperazone/Sulbactam	≥ 64	R	
Ceftazidime	≥ 64	R	
Ceftriaxone	≥ 64	R	
Cefepime	≥ 64	R	
Imipenem	4	R	
Ertapenem	2	R	
Aminoglycosides			
Gentamicin	8	I	
Amikacin	≤ 2	S	
Folate pathway inhibitors			sul3
Trimethoprim/ sulfamethoxazole	≥ 320	R	
Fluoroquinolone			mdtK
Ciprofloxacin	1	S	

MIC: minimal inhibitory concentration.

Our results demonstrate the capsular polysaccharide synthesis gene clusters in various strains of *R. planticola* and advance our understanding of the relationship between gene regions. Moreover, these findings may be useful for further development of genotyping in this organism. Also, the results of genome-wide prediction of multiple efflux systems and the comparative *in silico* study provide novel insights into the genome of GODA and lay the foundation for future experimental studies.

*Data availability* - This genome project has been deposited at the NCBI/GenBank (BioProject PRJNA375797), and includes the raw read data, assembly, and annotation. The assembly is available under accession CP019899; the version described in this paper is version CP019899.
